# Smoke and Alcohol Free with EHealth and Rewards (SAFER) pregnancy study: a before−after study protocol

**DOI:** 10.1038/s41533-020-00209-5

**Published:** 2020-11-18

**Authors:** Leonieke J. Breunis, Marlou L. A. de Kroon, Lyzette T. Laureij, Lieke de Jong-Potjer, Eric A. P. Steegers, Jasper V. Been

**Affiliations:** 1 Department of Obstetrics and Gynaecology, Erasmus MC—Sophia Children’s Hospital, Rotterdam, The Netherlands; 2 Department of Health Sciences, University Medical Center Groningen, University of Groningen, Groningen, The Netherlands; 3 Department of Paediatrics, Division of Neonatology, Erasmus MC—Sophia Children’s Hospital, Rotterdam, The Netherlands; 4 Department of Public Health, Erasmus MC, Rotterdam, The Netherlands

**Keywords:** Lifestyle modification, Preventive medicine

## Abstract

Despite existing interventions, tobacco smoking and alcohol consumption during pregnancy are common. The Smoke and Alcohol Free with EHealth and Rewards (SAFER) pregnancy intervention combines monthly group sessions, access to a web-based platform and incentives upon biochemically validated cessation for a maximum duration of 6 months to promote cessation of smoking and alcohol use before and during pregnancy. To inform development of the SAFER pregnancy intervention, two focus groups with the target population were held beforehand, with results reported here alongside the final SAFER pregnancy study protocol. In a before−after study we aim to include 66 women who are pregnant or have a wish to become pregnant and who smoke and/or consume alcohol (i.e. target population of the SAFER pregnancy intervention). The primary outcome measure is cessation of smoking and/or alcohol use at 34−38 weeks of gestation, or after six group sessions if women did not become pregnant during the study period. Secondary outcomes focus on the barriers and facilitators for implementation of the SAFER pregnancy intervention.

## Introduction

Smoking during the preconception period and/or pregnancy is associated with many adverse health outcomes. It decreases fertility and increases the risk of an ectopic pregnancy or miscarriage^1^. In addition, smoking during pregnancy increases the risk of adverse perinatal health outcomes, such as being small for gestational age, preterm delivery, birth defects, and even perinatal death^2,3^. Children born to mothers who smoke are also more likely to develop asthma, obesity, and respiratory infections, and are more likely to take up smoking later on^4–6^. Despite these risks, only half of women who smoke, successfully quit smoking because of their pregnancy or planning of pregnancy. As such, in the European Region, 5.9% of pregnant women are daily smokers^7^. In the Netherlands 3.5% of pregnant women smoke throughout their entire pregnancy^8^. Risk factors for continuation of smoking during pregnancy include having an unplanned pregnancy, having a partner who smokes, and a low socio-economic status (SES)^9,10^. Alcohol is another toxic substance that is commonly used and its consumption is socially acceptable in many cultures. Alcohol consumption during pregnancy is associated with an increased risk of miscarriages, fetal growth restriction, preterm delivery and Fetal Alcohol Spectrum Disorders (FASD)^11,12^. FASD include a range of (developmental) disorders and constitute a major cause of preventable intellectual disability in children^13^. The prevalence of alcohol consumption during pregnancy is estimated at 25% in Europe and 4.2% in the Netherlands^8,14^; the global prevalence of FASD is estimated at 2.3%^15^. Risk factors for continuation of alcohol use during pregnancy include a low educational level, a young maternal age, frequent alcohol use before pregnancy and having a depression^16–18^. Importantly, smoking and alcohol use during pregnancy often co-occur resulting in cumulating health risks^18,19^.

Although a number of interventions successfully promote smoking cessation during pregnancy, with relative risks for smoking cessation ranging from 1.25 for less intensive interventions to 1.44 for counselling, these are effective in only a minority of pregnant women^20,21^. The evidence concerning promotion of abstinence of alcohol during pregnancy is very limited^22^. This indicates that there is a need to continue developing more effective interventions tailored at pregnant women and women with a wish to conceive who continue to smoke or use alcohol. Combining various existing interventions or successful elements of interventions can potentially optimise the effectiveness of smoking cessation interventions^20,21^.

Recent studies indicate that provision of financial incentives (i.e. rewards for a specific goal with the purpose to motivate) can be effective for inducing behavioural change, including smoking cessation^23,24^. Research on incentives for alcohol cessation is scarce and has only been conducted among non-pregnant participants, with variable results. One small study in non-pregnant adolescents was unable to demonstrate a significant impact of incentives on 36-week abstinence (odds ratio 1.21 [95% CI 0.38–3.85])^25^. Two other studies in heavy drinking people showed that incentives prolonged participation in the intervention (84% versus 22% for 8-week intervention, *p* value < 0.001 (chi-square test))^26^ and abstinence (69% versus 39% completed 8-week intervention, *p* value < 0.05 (Breslow comparison) and 8.0 versus 2.9 consecutive days of abstinence, *p* value < 0.05 (multiple regression analysis))^26,27^.

Attending group sessions to increase knowledge, social support and health literacy, and eHealth-based interventions such as a web-based platform, are also promising for reducing smoking among women who are either pregnant or planning pregnancy, with relative risks for smoking cessation ranging from 1.21 for social support to 3.06 for eHealth-based interventions; however, many women continue to smoke during pregnancy^20,28^. The limited evidence concerning promotion of abstinence of alcohol during pregnancy shows that health education is often part of successful interventions for behavioural change during pregnancy and social support is important in helping women to reduce alcohol use^29,30^. In addition, eHealth-based interventions concerning alcohol cessation during pregnancy have been shown to be effective in increasing alcohol abstinence during pregnancy (odds ratio varying between 2.77 and 4.72) in favour of the eHealth-based intervention^31,32^.

The effectiveness of a combination of incentives, group sessions and an eHealth-based intervention to reduce smoking and alcohol use has never been studied before, neither in pregnant nor in non-pregnant women.

In the Smoke and Alcohol Free with EHealth and Rewards (SAFER) pregnancy study, we will investigate whether the combination of group sessions, a web-based platform and provision of financial incentives upon validated cessation is effective in reducing smoking and alcohol use in women before and during pregnancy. Given the multiple novel aspects of the intervention, the primary aim is to assess the feasibility and acceptability of the SAFER pregnancy approach so as to potentially inform design of a larger randomised experiment ideally powered to assess changes in pregnancy outcomes. The innovative multifaceted intervention has been developed in close collaboration with the target population. In addition to presenting the protocol for the SAFER pregnancy study, here we also report results from the focus group study with members of the target population that was undertaken prior to the SAFER pregnancy study so as to inform the development of the SAFER pregnancy intervention.

## Methods

The SAFER pregnancy study is an uncontrolled before−after study embedded in primary care, designed to assess changes in smoking and alcohol use among pregnant women and women with a wish to conceive following a complex intervention consisting of group sessions, access to a web-based platform, and provision of incentives upon validated smoking and alcohol cessation. The SAFER pregnancy study has been registered in the Netherlands Trial Register, reference NL7493 on 4 February 2019 (https://www.trialregister.nl/trial/7493). In preparation of the SAFER pregnancy study, we organised focus groups with the target population to inform the development of the SAFER pregnancy intervention. We discussed content of the group sessions, the nature and timing of the incentives, and the potential ways of recruitment of participants. The methods and results of this focus group study are described below, followed by a more detailed description of the SAFER pregnancy intervention.

### Focus group study: setting and participants

Two focus groups with the planned target population of the SAFER pregnancy study were held in February and April 2018. Potential participants could be referred by healthcare providers (e.g. midwives, gynaecologists and general practitioners) working in the municipality of Zoetermeer, a city in the Netherlands, or register themselves following recruitment via promotion material in local newspapers and social media if they were interested in participation. Women were eligible if: (1) they were planning pregnancy, were pregnant, or recently gave birth, (2) were over 18 years old, and (3) gave written permission for audiotaping of the focus group. Women were excluded if they insufficiently understood the Dutch language. The first focus group consisted of women who smoked at least one cigarette a day or drank at least five units of alcohol per week during pregnancy or while planning pregnancy. The second focus group consisted of women who had successfully quit smoking or using alcohol because of their pregnancy or planning of pregnancy. We intended to include 8−12 women in each focus group.

### Focus group study: data collection

Prior to the focus group study, participants received the statements that were discussed during the focus group study (Supplementary Table 1) and a questionnaire assessing personal characteristics by e-mail. The statements were grouped into: knowledge, existing support, and future support. Because extensive research on eHealth-based interventions for addressing smoking and alcohol use in the target population already existed, this topic was not discussed in the focus groups^28,31–38^. The questionnaire addressed smoking and alcohol use by the participants and their partners, whether they were already pregnant and whether they had a Western or non-Western background. The focus group study was conducted by three researchers (two at each focus group): L.J.B. guided the focus groups; M.L.A.d.K. and L.T.L. took notes. The researchers did not know the participants beforehand. The focus groups were conducted in Zoetermeer and lasted a maximum of 2 h.

Each focus group started with an introduction explaining the aim of the study and reassuring confidentiality. Both focus groups were audiotaped. All participants received a gift voucher worth 25 euros.

### Focus group study: data processing and analysis

All transcripts were transcribed verbatim, anonymised and returned to the participants for member checking. Prior to analysis, L.J.B. developed a coding scheme based on the framework of Fleuren et al., which states that dissemination, adoption, implementation, and continuation are the four consecutive stages within an innovation process^39^. According to this framework, transition of one stage to the next may be affected by the end user, the innovation itself, the socio-political environment, and by characteristics of the organisation^39^. Therefore, each of these factors should be taken into account when developing, implementing, and evaluating interventions. We used this framework approach with thematic content analysis to broaden our focus beyond evaluating aspects of the innovation (the SAFER pregnancy intervention) solely. By taking this approach we ensured that other aspects of the intervention that may affect feasibility and acceptability were also taken into account^40^. Before analysing the focus group interviews, subthemes were defined for each factor (for example for the factor ‘end user’, transcripts could be coded as emotions, knowledge, etc.). The factor ‘innovation’ consisted of multiple components since the SAFER pregnancy intervention combines multiple existing interventions. There was a different subheading within this factor for each component of the intervention, except for eHealth as stated before. During the analysis, some subthemes were added. The complete coding scheme is shown in Supplementary Table 2. Transcripts were independently coded by L.J.B. and L.T.L. If disagreement occurred, codes were discussed with a third researcher (M.L.A.d.K.) until consensus was reached. We used the qualitative software programme NVivo (QSR International Ltd. Version 12) to support data analysis.

### Focus group study: results

Participant recruitment proved challenging. Thirty-three women were referred to the researchers; six women could not be reached despite several phone calls and e-mails, and seven refrained from participation. Of the remaining 20 women, 14 agreed to attend one of two focus group interviews; the other women could not make it on the scheduled date and time. Five of the 14 women however did not show up. Eventually, focus group 1 consisted of five women: three were pregnant and two had a wish to conceive. Focus group 2 consisted of four women: one had a wish to conceive, one was pregnant and two recently gave birth. All nine women participated because of their smoking behaviour; one woman had experienced minor issues quitting alcohol use. Participant characteristics are shown in Supplementary Table 3.

A summary of the lessons learned from the focus group study that we implemented in the SAFER pregnancy intervention is shown in Table 1. Detailed results and illustrative quotes are shown in Supplementary Table 4. In summary, participants regarded it is important to understand the perspectives of women who smoke before and during pregnancy and understand the difficulties they face when trying to quit smoking. A strong motivation is important, but women report that this motivation decreases when the smoking behaviour is addressed in a negative way or when healthcare providers are perceived to ‘force’ cessation. Women who successfully quit smoking indicated that it is best to make a plan on how to quit: set a quit date, discuss potential use of nicotine replacement therapy and plan how to deal with cravings and boredom. During cessation, women stated that support in difficult moments should be easily accessible, preferably 24/7. Women felt that during the group sessions tips and tricks about smoking cessation should be shared, whereas discussing information concerning a healthy lifestyle more generally was undesirable. Incentives were not believed to increase motivation for smoking cessation but were believed to support within the process of quitting. The type of incentives should be discussed beforehand and women should have an option to choose which incentive they wish to receive. Furthermore, most women preferred multiple smaller incentives at brief intervals over larger ones at longer intervals. It was also felt stop-smoking-interventions should be available at low or even no costs.

**Table 1:** Summary of the lessons of the focus group study that were implemented in the design (detailed results and illustrative quotes are shown in Supplementary Table 4).

Part of SAFER pregnancy intervention	Implementation in the SAFER pregnancy intervention
Home visit and quit plan	Set a quit date, identify potential difficult moments, plan distraction and discuss the habit of smoking and dealing with habits. Discuss how to deal with relatives who smoke and do not smoke. Discuss the option of using nicotine replacement therapy during pregnancy.
Group sessions	Only focus on women who are pregnant or want to become pregnant. Each session is a standalone session so women who already quit smoking share their experiences with women who did not quit smoking yet. Exchange tips and tricks and discuss nicotine replacement therapy. Discuss hazards of smoking and alcohol consumption before and during pregnancy during an interactive and informative session. Focus group sessions on dealing with stress, distraction from smoking, identity, and diet. Let the informative session be guided by an experienced lifestyle coach who knows different ways of cessation.
Online platform	Actively refer participants to an online platform that provides help 24/7.
Incentives	Let women choose which reward they prefer for cessation. Provide monthly incentives instead of one large incentive over a longer period of time. Make the value of the incentives larger over time to motivate quitting early during pregnancy and remaining abstinent over a longer period of time.
Validation	Only provide incentives after biochemical validation.
Costs	Participation should be free.
Other	Give information on cessation support for partners.

Due to the low number of participating women, data saturation might not have been reached. Given the difficulty in recruiting women for the focus groups and in the light of the relevant information collected to inform the design of the SAFER pregnancy study, we pragmatically decided to proceed with finalising the development of the intervention and to start piloting it.

### SAFER pregnancy study: study design

The SAFER pregnancy study is a prospective, uncontrolled before−after study.

### SAFER pregnancy study: setting and participants

The study will be conducted in Zoetermeer, Benthuizen (small village that affiliated its care with Zoetermeer) and Rotterdam, the Netherlands. Previous research indicated that the municipality of Zoetermeer (approximately 125,000 inhabitants and 1300 livebirths annually) had the highest perinatal mortality in the Netherlands^41^, and it has a relatively large deprived population with likely clustering of risk behaviours such as smoking and alcohol use. Within Rotterdam (approximately 640,000 inhabitants and 7900 livebirths annually), more than half of children grow up in a neighbourhood with low SES and one in five children grow up in poverty^42^.

Women are eligible if:

- pregnant or having a wish to become pregnant within 6 months;
- smoking at least one cigarette a day and/or drinking at least three units of alcohol a week.

Exclusion criteria are:

- less than 18 years of age;
- more than 20 weeks pregnant;
- insufficient mastery of the Dutch language;
- unwilling to undergo urinary, and/or breath testing (when reporting smoking), and/or blood testing (when reporting drinking alcohol).
- urinary cotinine level below 50 µg/L, carbon monoxide (CO) level <7 parts per million (ppm) (when reporting smoking) or PhosphatidylEthanol (PEth) test below 7 µg/L (when reporting drinking alcohol) at inclusion;
- use of hard drugs.

Eligible women will be informed about the study by their midwife, obstetrician, primary care physician, healthcare provider of the outpatient clinic ‘Achieving a Healthy Pregnancy’ of the Erasmus MC or other healthcare providers (e.g. physiotherapist, physician at the centre for youth and family). The Erasmus MC (University Hospital in Rotterdam) provides an outpatient clinic (‘Achieving a Healthy Pregnancy’) for couples with a wish to conceive. This outpatient clinic focuses on improving healthy behaviour to promote a (healthy) pregnancy, and to prevent adverse health outcomes for mother and child. Healthcare providers of Zoetermeer, Benthuizen and the outpatient clinic are engaged in the study via regular meetings, newsletters and regular personal contact with the study team by phone and e-mail. If women are interested in participation, the healthcare provider will send their contact information to the researcher. In addition, participants will be recruited through promotion material (e.g. posters at schools, in waiting rooms and centres for youth and family and advertisement in local newspapers). Within 1 week, the researcher will contact the potential participant via telephone and inform her about the study and, if she is still interested in participating, make an appointment for a home visit.

Information about the study and the informed consent form (ICF) are sent by e-mail to the potential participant. During the home visit the study will be further explained, the ICF will be signed, biochemical validations (urinary cotinine level, CO breath test and/or PEth test to validate smoking and/or alcohol use among potential participants) will be performed as indicated, and a cessation plan will be made with the researcher (L.J.B.). A quit date will be set within 2 weeks after the home visit, potentially difficult moments will be discussed, and a plan to avoid or deal with these moments will be devised by the participant. Using motivational interviewing, motivation and self-confidence will be increased.

### SAFER pregnancy study: intervention

Based on existing literature and the focus group study we developed the SAFER pregnancy intervention, which consists of a web-based platform, group sessions and provision of incentives upon validated cessation of smoking and alcohol use in addition to care as usual. All components are described in detail below.

Participants will actively be directed towards an existing web-based platform: Smarter Pregnancy (www.slimmerzwangeronderzoek.nl). Smarter Pregnancy has been demonstrated to improve nutrition and lifestyle behaviour and is reimbursed by many Dutch health insurers^33^. For our study, the platform will be made freely available and its use will be encouraged. This platform maps the medical and non-medical (e.g. type of food intake, substance abuse, physical activity) risk profile of participants via online surveys. Based on this information it stimulates adoption of a healthy lifestyle using personalised e-mails. These contain tips, facts and recommendations to promote healthy behaviour and healthy food recipes.

Participants will be allocated into groups (3−10 participants per group), preferably based on the neighbourhood they live in. Participants will join monthly group sessions with a maximum of six sessions. The group sessions aim to provide peer support, increase self-efficacy, stimulate avoiding risk behaviour, and adopt a healthy lifestyle by providing information and organising activities concerning a healthy diet, sport activities, and cessation of smoking and alcohol use. There is no particular sequence of sessions; thus, women can enter the study at any time point.

Each session will take approximately 2 h. The first half hour of each session will be used to introduce participants to each other and to share experiences of the cessation process. The next 1.5 h will consist of other activities as described below. A study team member will be present at each group session.

- Sport session: Two of the six sessions (with two other sessions between them) will consist of yoga and power walking. The activities are supervised by instructors with specific expertise in working with pregnant women.
- Informative session: One group session will be used to interactively inform participants about the disadvantages of smoking and drinking alcohol and the advantages of cessation. This will be discussed using quizzes, and sharing of experiences and knowledge. This session will be led by a lifestyle coach.
- Identity session: Behaviour and someone’s identity are closely interrelated; identity can influence behaviour^43^, but people may also base their identity on their behaviours^44^. Smokers with a strong non-smoker identity more often attempt to quit smoking, and transition from a smoker identity towards a non-smoker identity may be necessary for successful quitting^45,46^. Also, moral identity as a mother is an important motivator for smoking cessation during pregnancy^47^, and transition to a non-smoking mother identity seems important in relapse prevention^48^. Drinking identity predicts problem drinking among college students^49,50^, but the link between a strong drinking identity and alcohol cessation has not been studied in relation to pregnancy. Whether strengthening the good mother identity affects smoking and alcohol drinking cessation is unknown. In this group session participants will explore their current and future identities in relation to smoking, alcohol and becoming a mother. Participants will be asked to search for images matching their ideas concerning their current and future identities. In addition, they will be asked to write a short text about their future self. The underlying idea is that participants may elicit positive attributes when considering their future non-smoking/non-drinking selves as opposed to them sustaining their risk behaviours. This may be expected to provide internal motivation to support cessation.
- Adaptive content session: For this session participants may choose from one of different subjects: a creative session (knitting or crocheting led by an arts facility); a vocal session (voice liberation or singing workshop, led by a singing teacher); mindfulness (led by a mindfulness trainer); or an informative session about pregnancy and the postpartum period (led by a midwife and maternity care organisation).
- Cooking session: During this session, participants will prepare and enjoy a healthy meal together. During the preparation participants will receive information concerning healthy ingredients and the do’s and don’ts of healthy cooking.

Participants are eligible to receiving incentives after biochemically validated cessation of smoking and/or alcohol use. We used an existing ethical framework when designing the various aspects of providing incentives in this study^51^. During each group session, participants who indicate that they quit smoking or drinking alcohol are invited to have this biochemically validated via a CO breath test (smoking) and/or PEth test (alcohol use). As soon as cessation is validated, the participant will receive the incentive. Incentives will be provided both at the individual and at the group level. To decrease the costs of the intervention and to increase local involvement, local entrepreneurs will be approached to provide incentives in the form of, for example, vouchers to locally support the project.

Individual incentives are vouchers to be spent at local businesses such as supermarkets, baby shops, or beauty salons. Tobacco products and alcoholic beverages are excluded. The monetary value of each incentive is listed in Table 2. The longer cessation is sustained, the more valuable the reward will be. When a participant relapses, the value of the next incentive will be reset. If a woman both smokes and uses alcohol, she is amendable to receiving incentives for cessation in both areas. Cessation of either smoking or drinking is followed by provision of ‘regular’ incentives, cessation of both is followed by incentives representing a monetary value of 1.5 times the ‘regular’ incentives (Table 2).

**Table 2:** Value of the incentives participants of the SAFER pregnancy study will receive per occasion when cessation of smoking and/or alcohol use has been achieved.

Consecutive number of times biochemically verified cessation^a^	Value of the incentive in euros if smoking or alcohol use are quit	Value of the incentive in euros if smoking and alcohol use are quit
1	15	22.50
2	20	30
3	20	30
4	30	45
5	40	60
6	60	90
Total	185	277.50

^a^During each group session, participants who indicate that they quit smoking or drinking alcohol are invited to have this biochemically validated via a CO breath test (smoking) and/or PEth test (alcohol use). As soon as cessation is validated, the participant will receive the incentive.

In addition to the individual incentives, group incentives will be provided to support a local neighbourhood improvement project (e.g. playground upgrade), which will be selected and developed by participants during the group sessions. Allocating incentives at the group level may be expected to promote feelings of shared responsibility and as such may be an additional extrinsic motivator for cessation of risk behaviour. The monetary value of the group incentives is comparable to that of the individual incentives (Table 2).

### SAFER pregnancy study: outcomes

**Fig. 1:**
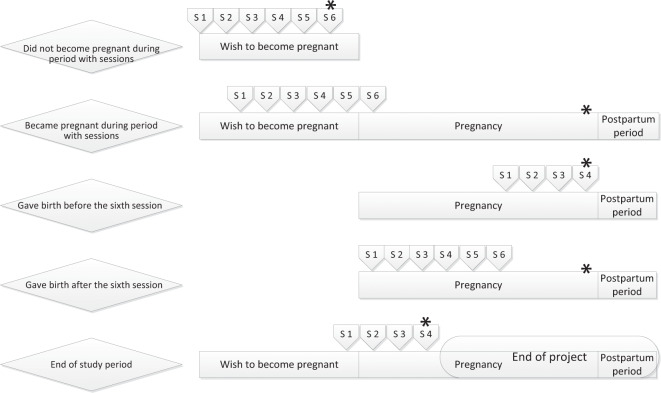
Main study endpoints in relation to a participant’s pregnancy status*. *Primary study endpoints are marked with an asterisk. S1−S6 = sessions 1−6.

The primary outcome is biochemically validated cessation of smoking or alcohol use at (Fig. 1):

- week 34−38 of gestation (if pregnant at inclusion or became pregnant during participation); or
- the end of the project period if <34 weeks pregnant at the time; or
- the last validation after six group sessions in those who did not become pregnant during participation.

The secondary outcomes are listed in Table 3. We will collect costs at the individual level of running the programme including the web-based platform and the group sessions to estimate costs per successful quitter. Using estimates from existing meta-analyses on the primary adverse outcomes associated with the risk behaviour that has been avoided by the SAFER pregnancy approach, we will furthermore calculate crude estimates of potential cost-effectiveness.

**Table 3:** Secondary study parameters/endpoints.

Data group	Data item	Data source
Barriers and facilitators of implementation (process measures)	How many sessions were attended by each participant	Study log
	How often participants logged in on the web-based platform	Smarter Pregnancy
	How many biochemical assessments were performed and the results of each assessment	Study log and lab results
	How many incentives were issued per person and in total	Study log
	How many local entrepreneurs were contacted and how many agreed to provide incentives	Study log
Perceived efficiency and appreciation of the web-based platform, the group sessions, and incentives	Experienced peer support and appreciation of the platform, the group sessions, and the incentives	Questionnaires
	Health behaviour before and during the study and after delivery	Questionnaires
	Reasons why lifestyle changes succeeded or failed	Questionnaires
	Evaluation of the group sessions	Questionnaires
Costs	The number of participants receiving incentives	Study log
	The amount of incentives received per participant	Study log
	The number of participants who achieve sustained cessation	Study log
Pregnancy outcomes	Date of delivery, singleton or multiple birth, birth weight, gestational age, gender, place of birth (e.g. hospital, home), type of delivery (e.g. caesarean section), Apgar score, admission to the Neonatal Intensive Care Unit (NICU), congenital abnormalities, mortality	Letter for the general practitioner if the participant consents, otherwise with a questionnaire
Identity changes	Identity factors associated with smoking status and/or drinking status	Questionnaire

### SAFER pregnancy study: data collection

Participants receive questionnaires through e-mail and fill them out online. They receive a voucher worth 15 euros if they filled out the questionnaire.

Questionnaire items are listed in Table 4. Participants receive a questionnaire at inclusion and 1 week before each group session. Women who are pregnant during the study period also receive a questionnaire at 34−38 weeks gestation and a final questionnaire 1 week postpartum. Women who do not become pregnant during the study period receive their final questionnaire after the sixth group session. In addition, a log on patient recruitment, collaboration with local entrepreneurs, and contact with healthcare providers and participants will be kept by the research team to explore barriers and facilitators for implementation of the intervention. In the log quantitative data will be noted, such as the number of potential participants referred and included and the amount of incentives provided (outcome measures are shown in Table 3). In addition, participants will fill out questionnaires about their experiences with the intervention and study itself during their participation and at the end of their participation. These data will give insight into acceptability and feasibility of the SAFER pregnancy intervention and therefore potential barriers and facilitators for proper implementation can be identified. These potential barriers and facilitators will be further qualitatively evaluated in a focus group study at the end of the SAFER pregnancy study, with the aim to evaluate the acceptability and feasibility of the intervention, in three separate focus groups with participants, leaders of the group sessions and involved healthcare providers.

**Table 4:** Data collection with questionnaires.

Data group	Data item
Baseline characteristics	Age
	Parity
	Ethnicity
	Household income and financial problems
	Educational level
	Marital status and relational problems
	Due date
	Lifestyle behaviour (smoking, alcohol use, diet, activity, drugs)
	Statements concerning lifestyle changes according to the Attitude—Social influence—Self-efficacy (ASE)-model
	Statements concerning identity
Level of addiction	Age started smoking
	Number of cigarettes smoked per day
	Smoking partner
	Intention and motivation to quit
Obstetric characteristics	Use of birth control
	Number of living children
	Number of miscarriages/stillbirths
	Number of premature births
	Number of children born small for gestational age
	Number of children born with congenital abnormalities
	Number of deceased children
Identity	Different statements concerning smoking, alcohol use and motherhood

At inclusion, smoking will be confirmed with a hand-held CO monitor (Micro + smokerlyzer)^52^ and a urinary cotinine test^53^, and alcohol use via a PEth^54,55^ test in 10 mL of blood. The cut-off value to identify active (as distinguished from passive) smoking in our study will be 7 ppm, in accordance with the NICE guideline^56^. Urinary cotinine values above 50 µg/L will be considered indicative of active (distinguished from passive) smoking. Whole blood PEth values above 6 µg/L will be considered indicative of alcohol use in the previous 2 weeks. If participants report smoking cessation during the study period, this will be validated with the CO test. At the primary endpoint, smoking cessation will be validated with the CO test and the urinary cotinine test. Cessation of alcohol will be validated with the PEth test.

### SAFER pregnancy study: data analysis plan

The proportion of women who quit smoking or alcohol use at the primary endpoint will be calculated as the number of women that have quit at this endpoint divided by the total number of women included in the study. Furthermore, we will use (backward) logistic regression analysis to explore independent predictors of effectiveness of the intervention. Descriptive statistics will be used for the secondary outcomes and results will be reported in narrative and tabular form. Open questions within the questionnaires will be evaluated qualitatively using thematic content analysis.

### SAFER pregnancy study: sample size calculation

Because vulnerable populations, as our target population, are difficult to reach and recruit for participation in medical research^57,58^ and because we aim to develop an intervention fit for the target population, we decided at this stage to pragmatically perform an uncontrolled before−after study. The overall aim is to obtain an estimate of potential effectiveness of the SAFER pregnancy intervention, while assessing its feasibility and acceptability. As such, findings from this study are ideally placed to inform design of a potential larger randomised experiment. The primary outcome is the proportion of women attaining sustained cessation of alcohol use and/or smoking. Because little research on interventions for alcohol cessation during pregnancy exists and only a small number of women were referred to the focus group study due to alcohol consumption, we based our sample size calculation primarily on smoking cessation. In a recent randomised controlled trial assessing the effectiveness of incentives to promote smoking cessation during pregnancy, 22.5% of the women in the intervention group reached the primary endpoint of cessation, versus 8.6% in the control group^59^. Accordingly, we set our true proportion to 25% and our null hypothesis proportion to 9%. With a power of 90% and a one-sided alpha of 2.5%, our intervention group would have to contain 49 participants in order to assess effectiveness of the SAFER pregnancy intervention versus the counterfactual scenario. Considering an expected dropout of 25%, we aim to include 66 women, who smoke and/or consume alcohol, at baseline.

### SAFER pregnancy study: ethics and dissemination

The Medical Research Ethics Committee of the Erasmus MC approved the study (NL67428.078.18). All participants will provide written informed consent. Each participant will receive a study number and all identifiable information of the participant and her baby will be stored separately from other data collected during the study. Only the core researchers (L.J.B., M.L.A.d.K. and J.V.B.) will have access to the identification list and the access passwords for both datasets. Study documents and data will be stored for 15 years after completion of the study.

### SAFER pregnancy study: safety

Due to the negligible risks of the SAFER pregnancy study, we will not collect or report adverse events and a Data Safety Monitoring Board will not be established. In concordance with the Guideline for Good Clinical Practice, the study will be monitored at least once a year by an independent and qualified monitor.^60^

## Discussion

Design of the SAFER pregnancy study was based on literature, guidelines, and results of the focus group study described here alongside the final protocol of the SAFER pregnancy study.

A major strength of the SAFER pregnancy intervention, embedded in existing primary care networks, is that it has been developed in close collaboration with the target population. Strengths of the focus group study in itself were that we identified barriers and facilitators for cessation by including women who continued to smoke and women who quit smoking during pregnancy in two different groups. In doing so, every participant was able to speak freely without the risk of feeling judged. Although we did not collect objective information regarding socio-economic indicators from focus group participants, many of them shared information during the discussions indicative of having a low socio-economic status (e.g. poor financial status, unemployment, single status, former drug abuse). Due to this, we believe the participants of the focus group study were representative for the target population of the SAFER pregnancy study. Limitations of the focus group study were the low number of participants and the fact that alcohol use was low among included women. Another strength of the SAFER pregnancy intervention is that it combines several interventions previously shown to be effective in promoting cessation of smoking and/or alcohol use. Also, the intervention addresses different aspects of addiction (e.g. identity, stress) and besides providing preliminary data on potential effectiveness this study we will assess facilitators and barriers to implementation of the intervention. Together the findings of the study may thus inform the design of a larger randomised trial to assess the impact of the SAFER pregnancy intervention on cessation and birth outcomes. Limitations of our study are that we will not have a control group and that, due to a lack of financial resources, we are unable to actively engage partners in the intervention.

The innovative SAFER pregnancy approach can potentially bring about substantial health benefits for mothers and children by addressing two important and common risk factors for adverse pregnancy outcomes which are entirely preventable.

### Reporting summary

Further information on research design is available in the Nature Research Reporting Summary linked to this article.

## Supplementary information

Supplementary Information

Reporting Summary

## Data Availability

We intend to provide an anonymised version of the dataset upon reasonable request.

## Code availability

Results of this study including the statistical code will be published in a peer-reviewed journal and be presented at (inter)national meetings.
