# Supplementary material for: Smoke and Alcohol Free with EHealth and Rewards (SAFER) pregnancy study: a before−after study protocol
Source: NPJ Prim Care Respir Med. 2020 Nov 18;30:51. doi: 10.1038/s41533-020-00209-5 (PMC7674488; doi:10.1038/s41533-020-00209-5)
Supplement: Supplementary file 1 — Supplementary Information [file 41533_2020_209_MOESM1_ESM.pdf]

Supplementary Table 1 Statements discussed during the focus groups (translated from Dutch)

**Focus group 1**	**Focus group 2**
Knowledge and attitude	
It is healthy for me and my future child to quit smoking before or during my pregnancy.	I quit smoking and/or drinking alcohol during pregnancy because this was better for my own health.
It is healthy for me and my future child to quit drinking alcohol before or during my pregnancy.	I quit smoking and/or drinking alcohol during pregnancy because this was better for the health of my future child.
	I quit smoking and/or drinking alcohol because peers/ family made me.
	I quit smoking and/or drinking alcohol because my healthcare provider (for example the general practitioner or midwife) urged me to.
Anyone can quit smoking.	Anyone can quit smoking.
Anyone can quit drinking alcohol.	Anyone can quit drinking alcohol.
Existing support	
Currently available support for smoking cessation is sufficient.	Current support for smoking cessation is sufficient.
Current support for alcohol cessation is sufficient.	Current support for alcohol cessation is sufficient.
If I want to quit smoking and/or drinking alcohol the support of my partner is very important.	I quit smoking and/or drinking alcohol on my own.
The general practitioner is sufficiently capable to determine whether a pregnant woman or	The general practitioner is sufficiently capable to determine whether a pregnant woman or

woman with a wish to conceive smokes or drinks alcohol.	woman with a wish to conceive smokes or drinks alcohol.
The midwife is sufficiently capable to determine whether a pregnant woman or woman with a wish to conceive smokes or drinks alcohol.	The midwife is sufficiently capable to determine whether a pregnant woman or woman with a wish to conceive smokes or drinks alcohol.
If I am not motivated to quit smoking/drinking alcohol it is of no use for people in my surroundings or my healthcare provider to discuss this with me.	If I am not motivated to quit smoking/drinking alcohol it is of no use for people to discuss this with me.
Future support	
A reward for smoking cessation before or during pregnancy would help me to quit smoking.	
A reward for alcohol cessation before or during pregnancy would help me to quit drinking alcohol.	
A reward for smoking and/or alcohol cessation should ideally be provided as cash.	
A reward for smoking and/or alcohol cessation should ideally be provided as vouchers for food supplies for me and my family.	
A reward for smoking and/or alcohol cessation should ideally be provided as vouchers for fun things that you otherwise would not do.	

Online information concerning smoking and/or alcohol consumption with extra tips would be helpful to quit smoking and/or drinking alcohol.	
It is important that support provision for smoking and/or alcohol cessation is free of charge.	It is important that support provision for smoking and/or alcohol cessation is free of charge.
It would be nice to quit smoking and/or drinking alcohol together with a group of pregnant women or women with a wish to conceive.	
Quitting smoking and/or drinking alcohol with a group of pregnant women or women with a wish to conceive will help me to quit smoking/drinking alcohol.	
I am willing to attend some sessions about smoking/alcohol cessation together with others to help me quit smoking and/or drinking alcohol.	
These sessions should be aimed at bonding with others and about a healthy lifestyle (information about healthy nutrition, smoking and alcohol cessation and sufficient exercise).	
These sessions should be aimed at smoking and/or alcohol cessation and no other subjects.	

Support for smoking and/or alcohol cessation before and during pregnancy should be anonymous.	
To make a reward for smoking and/or alcohol cessation possible, I am willing to take a breath test or blood test to prove that I have actually stopped smoking and/or drinking alcohol.	
It is useful to not only be provided a reward for smoking and/or alcohol cessation personally, but also to the group if the group has quit together.	
	Support for smoking cessation is really necessary to quit smoking, alone it is not possible.
	Support for alcohol cessation is really necessary to quit smoking, alone it is not possible.
	It is important that my partner is involved within the support for smoking and/or alcohol cessation.
	Rewarding people for smoking and/or alcohol cessation would motivate me to start smoking and/or drinking alcohol again.

Supplementary Table 2 Topic list used for coding of the transcripts of the focus group study

**Factor**	**Subtheme**
**End user**	Emotions
	Knowledge
	Self-efficacy
	Motivation
	Role of friends/family
	Coping mechanism
	Making a plan
	Habit/boredom
**Innovation – in general**	Timing
	Availability
	Other helpful parts/interventions
**Innovation – group sessions**	Willingness
	Content
	Leader
	Attendees
	Helpfulness
	Group pressure
**Innovation- incentives**	Willingness and motivation
	Type of reward
	Group reward

	Duration between rewards
	Value of rewards
	Chance of success
	After ending of reward
**Socio-political environment**	Costs
	Politics
**Organisation**	Involvement of healthcare provider
	Attitude of healthcare provider
	Information given by healthcare provider
	Willingness to perform biochemical validation
	Knowledge of healthcare provider

Supplementary Table 3 Sample demographics of the participants of the focus group study

ID	Focus group	Age (years)	Smoking behaviour	Alcohol consump-tion	Smoking partner	Alcohol consumption partner	Pregnancy	Ethnicity Western/non-western
P1	1	23	1-10 cig/day	Never	Yes	Never	1th trimester	Western
P2	1	30	1-10 cig/day	< 1 unit/week	Yes	Yes	Preconcep-tional	Non- Western
P3	1	39	>10 cig/day	Quitted	No partner	No partner	2th trimester	Missing
P4	1	29	>10 cig/day	< 1 unit/week	Yes	Yes	1th trimester	Western
P5	1	32	1-10 cig/day	Never	Quitted	Yes	Postpartum and preconcep-tional	Western
P6	2	49	Quitted	1-3 unit/day	Never	Yes	Postpartum	Western
P7	2	36	1-10 cig/day	< 1/week	Never	Never	Postpartum	Non- Western
P8	2	27	Quitted	Quitted	Quitted	Yes	3th trimester	Western
P9	2	25	Quitted	< 1 unit/week	Quitted	Yes	Postpartum	Western

Supplementary Table 4 Lessons of the focus group study

Factor	Lesson learned	Illustrative quotes	Implementation in the SAFER pregnancy intervention
**End user**	Women experience advantages of smoking (social occasion and relaxation) and disadvantages of smoking cessation (creates tension and irritation towards partner and other children, women might gain weight during cessation).	P3: “Yes, that is what my children are saying too: “You go and start smoking again, that might be better for you. You are cranky.”.”<br>P6: “Err, I found it annoying. Because I had to quit smoking and quit drinking alcohol. And that I found very unsociable.”<br>P7: “The only thing that err, at this moment, helps me, is smoking. And not just one, several cigarettes in succession. And then I am just relieved.”<br>P1: [After telling she had quit smoking for 1.5 years] “Then stressful things [come along] and yes, then you start smoking again.”	House visit/quit plan: Discuss perceived advantages of smoking/drinking and plan on how to attain those same advantages in a different way. Discuss the perceived disadvantages of cessation and plan approaches to avoid those disadvantages.<br><br>Group sessions: Discuss problems women experience while quitting and exchange tips and tricks.<br>Focus some group sessions on dealing with stress (e.g. the sport sessions and the adaptive content session).
	Women know smoking during pregnancy is	P2: [After telling she is attending a stop-smoking-	Group sessions: Discuss (often unknown) hazards of smoking

	unhealthy for their child. However, they underestimate the risks and do not always make the connection between a disease and smoking. Alcohol is considered as less dangerous for the unborn child.	outpatient clinic] “It is also like, I know that smoking is bad of course, so yes, then I think I will be sitting there and I will be hearing things that I already know myself.”<br>P4:”My sister quit smoking and she has a child with hidden reflux. Another friend of mine quit smoking, her child was in the hospital for a week after birth because of a fever and after that a hospital acquired bacteria and another friend of mine quit smoking and her child needed help pooping for multiple weeks. Then I think “I smoked, she smoked and another friend smoked [all during pregnancy] and they all have healthy children”, and that is also playing a role.”<br>P6: “Because I err, yes maybe the background information [about risks of alcohol during	and alcohol consumption before and during pregnancy during an interactive and informative session.

		pregnancy] is not as good [as with smoking].” … “And with smoking I had the idea that it came directly into the blood stream from the little one [unborn child].” [in contrary to alcohol]	
	Support to quit smoking from people around them is important for the women who want to quit smoking. A partner or family members who smoke make it more difficult to quit. However, family or friends who disapprove smoking makes someone smoke more.	P1: “Yes, and it doesn’t make it easy that we both smoke at my home, together. So, that is also difficult.”<br>P4: [After telling she did not smoke at her parents’ house] ”I think that if I stayed there for a week, I would not have smoked for a week.”<br>P8: “But I really should not have it [cigarettes] close by. Because if I, if I, as soon as I would buy a pack, I would [moving as if she smokes] one after another.”<br>P3: “But you should not be breathing down my neck saying “You must stop	<u>House visit/quit plan:</u> Discuss how to deal with relatives who smoke and how to communicate the needs of the participant towards their relatives who do not smoke.<br><br><u>Other:</u> Give information on cessation support for partners (standard care).[a]

		smoking." P5 replying: "No, then I'll only smoke more."	
	Smoking is also habitual, something to do when feeling bored and a way of rewarding yourself. Seeking distraction is a way of smoking less or to even quit smoking. In order to stop smoking it is best to make a plan on when to quit smoking and what to do as distraction or reward.	P5: "And then you just need five [cigarettes]." … "You get up in the morning, you eat breakfast, you smoke, then you go for lunch and then you smoke a cigarette, you eat dinner then you smoke a cigarette and before going to bed. Well, that's exactly five." P4: [After telling she hates doing the household] "Well whoop, a cigarette, because I reward myself that I folded it [the laundry] up." P9: "Yes but that remains [smoking cessation being difficult], you should keep yourself busy, you should not go sit on the coach moping…" P8: "And the month after we both had our birthday. So then I said "Ok, our birthdays, you know, with friends in the garden, and then it's over." So	<u>House visit/quit plan:</u> Set a quit date, identify potential difficult moments, plan distraction and discuss the habit of smoking and dealing with habits.<br><br><u>Group sessions:</u> Discuss the problems women experience while quitting with each other and exchange tips and tricks. During the group sessions focus on distraction from smoking (e.g. the adaptive content session).

		half way January, it was really like, the weekend we celebrated our birthday, so I bought packs of cigarettes, we smoked that in the two days after and then it [smoking] was finished.”	
	Everybody wants to quit smoking because of their pregnancy. The biggest motivation for cessation is the health of their unborn child, but also saving money was a motivator, the fact that they do not want their child to start smoking in later life and the fact that smoking is smelly. Women who do smoke during pregnancy feel guilty about their addiction towards their unborn child and are ashamed.	P9: [After telling the child of her sister in law died during pregnancy while she smoked during pregnancy]: “If I was her, I would have put that on myself like “If only I hadn’t smoked.” Then maybe…”<br>P4: “And also every time I try to put on paper how much I, we [she and her partner], would save [if they would not smoke]… between €400 and €600 per month…”<br>P5: “We all set the wrong example [to their children].”<br>P4: “If I calculate how much time I am smoking instead of spending on her [daughter], I	

		feel so guilty. And yet I keep doing it.” P4: “When I was pregnant of my first child, I only smoked at home, because apart from two friends, nobody knew I was smoking because I told everybody I quit. Even my parents, they still don’t know I smoked during that pregnancy.” P4: “If I come downstairs in the morning and he [her partner] smoked and he kisses me, I think “Damn, your mouth smells.”	
	Women said everybody can quit smoking but it is very tough, and one has to be ready and highly motivated. How to become ready remained unclear.	P5: “Yes, everybody can quit smoking. But you need to have a backbone and mine is similar to that of a mollusc.” P1: “Well, at first I smoked a pack [of cigarettes] a day and when I found out I was pregnant, I decreased this towards five cigarettes a day.	

		And then [quitting] those final cigarettes, they are hard."<br>P3: "It is possible, but you have to be determined."<br>P5: "But wanting to quit is one thing. But, you also have to be done with it [smoking] and be ready for it [quitting smoking]." … "Then you may want to, but if you have not reached that point of "Well I'm completely done with it", than you will not succeed."<br>[After asking how to become ready to quit] P5: "I don't know. I think it is just a moment"<br>P9: "… well yes, I think that anyone can quit smoking. Sure, but you should really have to want it, so yes."	
**Innovation – in general**	It is important that help is not only available during regular working hours, or	P2: [After telling that the quit-smoking outpatient clinic will call every two weeks] "Yes,	<u>Online platform:</u> Actively refer participants to an online platform that provides help

	only once every couple of weeks. There should be an option for getting help 24/7. Also, help should be easy to find.	but I smoke every three hours…" P4: "Yes, that is why I'm saying that I should be able to call you at the moment my body is screaming for a cigarette, that is the moment I should be able to call you." P9: "So you should also know what all the possibilities for support are to be able to find it."	24/7 and is part of the SAFER pregnancy intervention.
	Many pregnant women do not know they are able to use nicotine replacement therapy. They feel they need help and are looking for something that makes the cravings go away, such as complementary medicine, or being in a place where there is no possibility to smoke.	P5: "Yes, you know, I have seriously considered signing up to an addiction clinic." P5: "My mother has tried [quitting] with the help of hypnosis. She did quit for quite some time back then." P4: "But if you are pregnant you cannot use Champix or Tabex or nicotine patches or whatever, you just have to quit, plus you are a hormone bomb. And then I think "Well… I can't do it."."	<u>House visit/quit plan:</u> Discuss the ability to use nicotine replacement therapy during pregnancy. Refer to their healthcare provider if interested in using this therapy.<br><br><u>Group sessions:</u> Discuss nicotine replacement therapy (e.g. informative session).

**Innovation – group sessions**	Most women are happy about attending group sessions. This helps them realise that they are not the only ones who smoke during pregnancy and they like sharing their experiences. Also, they can support each other when trying to quit smoking. However, they do not think the sessions alone are enough to help them quit smoking. And some women felt group sessions did not fit them.	P4: "So I think it helps to stimulate each other in a way, but I don't think that if you do only that [the group sessions], I don't think that's enough to really quit."<br>P9: "If you don't like it [group sessions], then you shouldn't try that either."	<u>Group sessions: </u>Only focus on women who are pregnant or want to become pregnant so the intervention fits the target population instead of a more general population.
	During the group sessions women would like to receive information on different ways of quitting (such as with medication or complementary medicine), share tips and tricks and like to hear experiences of	P2: "It is always nice to hear from others how they quit smoking. How they did it…" "…If you hear that from different perspectives "Oh, maybe I can try that [smoking cessation] in that way too.""	<u>Group sessions:</u> Let women in different stages of quitting attend, so each session is a standalone session. Let women who already quit smoking share their experiences with women who did not quit smoking yet.

	women who did succeed in quitting. Some women underlined the need to create awareness about why someone smokes.<br><br>Information on the risks of smoking during pregnancy and a healthy diet are less desirable. However, some women would like to hear information on how not to gain weight while trying to quit smoking.	P4: "Yes, tips and tricks, that would definitely help."<br>P6: "And at the moment you become aware of that [on why you smoke], then you could tackle that part. … I think you should look for the underlying thought on smoking. Instead of looking for the reason why someone should stop [smoking] for something that is not there yet [bad health]."<br>P5: "But I think that, you shouldn't want to impose a healthy diet on someone who is already quitting smoking. You know, fine if there is guidance like "Okay, you stop smoking now, but make sure you don't stuff yourself with potato chips". Great if there is, or, that there is a suggestion on how not to stuff yourself."	Let the informative session be guided by an experienced lifestyle coach who knows different ways of cessation. Focus sessions on identity (topics such as why women started smoking and why remaining a smoker will be discussed) and diet (because some women pointed out their fear of gaining weight).
	The person leading the group session(s) should understand the target	P4: "It doesn't have to be that you [the group leader] have actually smoked, but you	Group sessions: The researcher (LB) was present at both focus group interviews

	population and emphasise smoking cessation is difficult.	really have to understand very well that smoking cessation is not a "just flip the button, you just have to do it."."<br>P5: "What I really like is that the person who guides you, eh yes, is not a goat wool sock type [Dutch expression for an unworldly person] who has never done anything wrong himself/herself, because that really doesn't help, you know."	and is therefore familiar with the problems the target population faces, is also present at each group session.
	It is important that the women attending the group session(s) are a good match to each other.	P5: [After telling she attended group sessions for drug rehab] "Yes, that maybe, if I had had a nice group during that time or a group I found a connection with, I don't know what I was missing there, then I might have had another experience…"	Group sessions: Only focus on women who are pregnant or want to become pregnant so the intervention is more fit to the target population instead of a more general population.
**Innovation-incentives**	Women were very divided over the type of reward.	P6: "Yes, what could help is that instead of ten days, you	Incentives: Let women choose which reward they want for

	Some preferred luxury items, others preferred vacations, and others wanted to buy groceries. However, they all preferred money over vouchers so they could choose what to spend it on. It is important that before trying to quit smoking, women know what kind of reward they will receive.	receive twenty days [maternity leave], after the delivery.”<br>P4: “But if I am very honest, I rather think that money would motivate me more than things.”<br>P4: “I want to spend my reward on something for myself, not on something daily.”<br>P5: “Yes, then yes, than you can give me a weekend away worth €200, but I rather have those €200 so I can buy groceries.<br>P1: “I think that the money, then you can choose yourself on what you want to spend it. I think that that is it yes. A weekend away or a holiday.”<br>P5: “And if I receive a voucher, it is not on my bank account and I do not see that it went from minus to plus in one go.”	cessation. Because of moral considerations we decided not to provide cash rewards.[51]

		P1: "I think it would work for me. But I do have to know what it will be."	
	The group incentive was not often discussed, but women seemed not very enthusiastic about it.	P5: [After explaining the idea of a group reward] "No, I'm too selfish for that."	<u>Incentives:</u> Despite advise against the group rewards, we decided to implement both individual incentives and group incentives. We hypothesise that these group incentives contribute to obtaining social cohesion and support to each other.
	Women prefer multiple smaller incentives at smaller intervals over one big incentive after a long period. Especially at the beginning when cessation is the hardest.	P5: "I certainly think when you are in the initial phase where it is very difficult, I think it is very motivating when someone comes to provide you with a share of your reward."<br>P4: "O that is still manageable. And you know that after two weeks, that urge is getting less and less…"	<u>Incentives:</u> Provide monthly incentives instead of one large incentive over a longer period of time. Make the value of the incentives larger over time to motivate quitting early during pregnancy and remaining abstinent over a longer period of time.
	It is important that the incentive is only provided	P4: [After telling that her parents said that they would	<u>Validation:</u> Use biochemical validation of cessation and

	after proven cessation, otherwise women will not believe they do not receive the incentive and they lose their motivation. Opinions about the value of the reward were diverse and mostly depending on the financial status and motivation to quit.	not pay for her driving lessons if she would start smoking before age 18, but even though she did start smoking before 18 and they still payed for the lessons] "Somehow you think "They won't take that from me."<br>P2: "I would not do it [quitting] for less than €200."<br>P4: "Because people with enough money think "Pff, €600 [like it is nothing]" and I think "€600?! [very enthusiastic], with that I can pay my last debts."	only provide incentives after validation.
**Socio-political environment**	High costs are large barrier for attending/applying for treatment for smoking cessation.	P3: "It would be more fun if those things [treatment for smoking cessation] were reimbursed."<br>P8: [Discussing nicotine replacement therapy] "Yes, and if you compare it with how much many smoking	<u>Costs:</u> Free participation. Unfortunately we did not have the financial possibilities to provide nicotine replacement therapy for free.

		costs, depending on how much you smoke per day of course, then it is actually cheaper. However, you have to pay it all at once, instead of every time you buy a pack [of cigarettes].	
**Organisation**	All women are willing to undergo a breath test to validate smoking cessation. A blood draw was a higher barrier, although nobody saw that as a reason not to quit smoking.	Interviewer: "Would you be willing to undergo a breath test every month to see whether you quit smoking?"<br>P3: "Yes"<br>P5: "Yes"<br>P4: "Absolutely"	Validation: Use biochemical validation of cessation.

[a] Unfortunately because of financial reasons, we do not have the possibility to offer the SAFER pregnancy intervention to partners.
