# Supplementary material for: Smoke and Alcohol Free with EHealth and Rewards (SAFER) pregnancy study: a before−after study protocol
Source: NPJ Prim Care Respir Med. 2020 Nov 18;30:51. doi: 10.1038/s41533-020-00209-5 (PMC7674488; doi:10.1038/s41533-020-00209-5)
Supplement: Supplementary file 2 — Reporting Summary [file 41533_2020_209_MOESM2_ESM.pdf]

nature research

Corresponding author(s): NPJPCRM-00958

Last updated by author(s): Apr 23, 2020

Reporting Summary

Nature Research wishes to improve the reproducibility of the work that we publish. This form provides structure for consistency and transparency in reporting. For further information on Nature Research policies, see Authors & Referees and the Editorial Policy Checklist.

Statistics

For all statistical analyses, confirm that the following items are present in the figure legend, table legend, main text, or Methods section.

n/a	Confirmed	
☐	☒	The exact sample size (*n*) for each experimental group/condition, given as a discrete number and unit of measurement
☐	☒	A statement on whether measurements were taken from distinct samples or whether the same sample was measured repeatedly
☐	☒	The statistical test(s) used AND whether they are one- or two-sided<br>*Only common tests should be described solely by name; describe more complex techniques in the Methods section.*
☐	☒	A description of all covariates tested
☒	☐	A description of any assumptions or corrections, such as tests of normality and adjustment for multiple comparisons
☒	☐	A full description of the statistical parameters including central tendency (e.g. means) or other basic estimates (e.g. regression coefficient) AND variation (e.g. standard deviation) or associated estimates of uncertainty (e.g. confidence intervals)
☒	☐	For null hypothesis testing, the test statistic (e.g. *F*, *t*, *r*) with confidence intervals, effect sizes, degrees of freedom and *P* value noted<br>*Give P values as exact values whenever suitable.*
☒	☐	For Bayesian analysis, information on the choice of priors and Markov chain Monte Carlo settings
☒	☐	For hierarchical and complex designs, identification of the appropriate level for tests and full reporting of outcomes
☒	☐	Estimates of effect sizes (e.g. Cohen's *d*, Pearson's *r*), indicating how they were calculated

Our web collection on statistics for biologists contains articles on many of the points above.

Software and code

Policy information about availability of computer code

Data collection	Questionnaires will be provided online using limesurvey and gemstracker.
Data analysis	For this study R and SPSS will be used. For the qualitative part of the study Nvivo will be used.

For manuscripts utilizing custom algorithms or software that are central to the research but not yet described in published literature, software must be made available to editors/reviewers. We strongly encourage code deposition in a community repository (e.g. GitHub). See the Nature Research guidelines for submitting code & software for further information.

Data

Policy information about availability of data

All manuscripts must include a data availability statement. This statement should provide the following information, where applicable:

- Accession codes, unique identifiers, or web links for publicly available datasets
- A list of figures that have associated raw data
- A description of any restrictions on data availability

Results of this study including the statistical code will be published in a peer-reviewed journal and be presented at (inter)national meetings. We intend to provide an anonymised version of the dataset upon request.

Field-specific reporting

Please select the one below that is the best fit for your research. If you are not sure, read the appropriate sections before making your selection.

☐ Life sciences ☒ Behavioural & social sciences ☐ Ecological, evolutionary & environmental sciences

Behavioural & social sciences study design

All studies must disclose on these points even when the disclosure is negative.

Study description	The SAFER pregnancy study is a prospective, uncontrolled before-after study. The study will have quantitative and qualitative data.
Research sample	Women who are pregnant or planning pregnancy and who smoke and/or consume alcohol.<br>Smoking during the preconception period and/or pregnancy is associated with many adverse health outcomes. Still many women continue to smoke or drink alcohol in this period. Several singular interventions have been proven effective in reducing smoking and alcohol use in a small number of pregnant women. Combining several existing interventions might be beneficial for these woman. Therefore we developed an intervention with group sessions, a web-based platform and incentives.
Sampling strategy	The primary aim is to assess feasibility and acceptability of the SAFER pregnancy intervention so as to potentially inform design of a larger randomised experiment ideally powered to assess changes in pregnancy outcomes. At this stage we therefore decided to pragmatically perform an uncontrolled before-after study. The primary outcome is the proportion of women attaining sustained cessation of alcohol use and/or smoking. Because little research on interventions for alcohol cessation during pregnancy exist and only a small number of women where referred to the focus group study due to alcohol consumption, we based our sample size calculation primarily on smoking cessation. In a recent randomised controlled trial assessing the effectiveness of incentives to promote smoking cessation during pregnancy, 22.5% of the women in the intervention group reached the primary endpoint of cessation, versus 8.6% in the control group. Accordingly, we set our true proportion to 25% and our null hypothesis proportion to 9%. With a power of 90% and a one-sided alpha of 2.5%, our intervention group would have to contain 49 participants in order to assess effectiveness of the SAFER pregnancy intervention versus the counterfactual scenario. Considering an expected dropout of 25%, we aim to include 66 women at baseline.
Data collection	Participants receive questionnaires through e-mail and fill them out online. They receive a voucher worth 15 euros if they filled out the questionnaire. Participants receive a questionnaire at inclusion and one week before each group session. Women who are pregnant during the study period also receive a questionnaire at 34 to 38 weeks gestation and a final questionnaire one week postpartum. Women who do not become pregnant during the study period receive their final questionnaire after the sixth group session. In addition, a log on patient recruitment, collaboration with local entrepreneurs, and contact with healthcare providers and participants will be kept by the research team to explore barriers and facilitators for implementation of the intervention. In the log quantitative data will be noted, such as the number of potential participants referred and included and the amount of incentives provided (outcome measures are shown in table 3). In addition, participants will fill out questionnaires about their experiences with the intervention and study itself during their participation and at the end of their participation. These data will give insight into acceptability and feasibility of the SAFER pregnancy intervention and therefore potential barriers and facilitators for proper implementation can be identified. These potential barriers and facilitators will be further qualitatively evaluated in a focus group study at the end of the SAFER pregnancy study, with the aim to evaluate the acceptability and feasibility of the intervention, in three separate focus groups with participants, leaders of the group sessions and involved healthcare providers.<br><br>At inclusion, smoking will be confirmed with a hand-held CO monitor (Micro+ smokerlyzer) and a urinary cotinine test,and alcohol use via a PhosphatidylEthanol (PEth) test in 10 mls of blood. The cut-off value to identify active (as distinguished from passive) smoking in our study will be seven ppm, in accordance with the NICE guideline. Urinary cotinine values above 50 µg/L (corrected for creatinine) will be considered indicative of active (distinguished from passive) smoking. Whole blood PEth values above 6 µg/L will be considered indicative of alcohol use in the previous two weeks. If participants report smoking cessation during the study period, this will be validated with the CO test. At the primary endpoint, smoking cessation will be validated with the CO test and the urinary cotinine test. Cessation of alcohol will be validated with the PEth test.
Timing	We started this study in March 2019 and because this paper is a protocol, we are still collecting data.
Data exclusions	We did not complete our data collection yet and therefore there aren't any exclusions yet.
Non-participation	We did not complete our data collection yet and therefore we cannot give exact drop-out numbers yet.
Randomization	This is an uncontrolled before-after study and not randomisation took place.

Reporting for specific materials, systems and methods

We require information from authors about some types of materials, experimental systems and methods used in many studies. Here, indicate whether each material, system or method listed is relevant to your study. If you are not sure if a list item applies to your research, read the appropriate section before selecting a response.

Materials & experimental systems

n/a	Involved in the study
☐	☐ Antibodies
☐	☐ Eukaryotic cell lines
☐	☐ Palaeontology
☐	☐ Animals and other organisms
☐	☒ Human research participants
☐	☒ Clinical data

Methods

n/a	Involved in the study
☐	☐ ChIP-seq
☐	☐ Flow cytometry
☐	☐ MRI-based neuroimaging

Antibodies

Antibodies used: *Describe all antibodies used in the study; as applicable, provide supplier name, catalog number, clone name, and lot number.*

Validation: *Describe the validation of each primary antibody for the species and application, noting any validation statements on the manufacturer's website, relevant citations, antibody profiles in online databases, or data provided in the manuscript.*

Eukaryotic cell lines

Policy information about cell lines

Cell line source(s): *State the source of each cell line used.*

Authentication: *Describe the authentication procedures for each cell line used OR declare that none of the cell lines used were authenticated.*

Mycoplasma contamination: *Confirm that all cell lines tested negative for mycoplasma contamination OR describe the results of the testing for mycoplasma contamination OR declare that the cell lines were not tested for mycoplasma contamination.*

Commonly misidentified lines (See ICLAC register): *Name any commonly misidentified cell lines used in the study and provide a rationale for their use.*

Palaeontology

Specimen provenance: *Provide provenance information for specimens and describe permits that were obtained for the work (including the name of the issuing authority, the date of issue, and any identifying information).*

Specimen deposition: *Indicate where the specimens have been deposited to permit free access by other researchers.*

Dating methods: *If new dates are provided, describe how they were obtained (e.g. collection, storage, sample pretreatment and measurement), where they were obtained (i.e. lab name), the calibration program and the protocol for quality assurance OR state that no new dates are provided.*

☐ Tick this box to confirm that the raw and calibrated dates are available in the paper or in Supplementary Information.

Animals and other organisms

Policy information about studies involving animals; ARRIVE guidelines recommended for reporting animal research

Laboratory animals: *For laboratory animals, report species, strain, sex and age OR state that the study did not involve laboratory animals.*

Wild animals: *Provide details on animals observed in or captured in the field; report species, sex and age where possible. Describe how animals were caught and transported and what happened to captive animals after the study (if killed, explain why and describe method; if released, say where and when) OR state that the study did not involve wild animals.*

Field-collected samples: *For laboratory work with field-collected samples, describe all relevant parameters such as housing, maintenance, temperature, photoperiod and end-of-experiment protocol OR state that the study did not involve samples collected from the field.*

Ethics oversight: *Identify the organization(s) that approved or provided guidance on the study protocol, OR state that no ethical approval or guidance was required and explain why not.*

Note that full information on the approval of the study protocol must also be provided in the manuscript.

Human research participants

Policy information about studies involving human research participants

Population characteristics: See above

Recruitment	Eligible women will be informed about the study by their midwife, obstetrician, primary care physician, healthcare provider of the outpatient clinic 'Achieving a Healthy Pregnancy' of the Erasmus MC or other healthcare providers (e.g. physiotherapist, physician at the centre for youth and family). The Erasmus MC (University Hospital in Rotterdam) provides an outpatient clinic ('Achieving a Healthy Pregnancy') for couples with a wish to conceive. This outpatient clinic focuses on improving healthy behaviour to promote a (healthy) pregnancy, and to prevent adverse health outcomes for mother and child. Healthcare providers of Zoetermeer, Benthuizen and the outpatient clinic are engaged in the study via regular meetings, newsletters and regular personal contact with the study team by phone and e-mail. If women are interested in participation the healthcare provider will send their contact information to the researcher. In addition, participants will be recruited through promotion material (e.g. posters at schools, in waiting rooms and centres for youth and family and advertisement in local newspapers). Within one week, the researcher will contact the potential participant via telephone and inform her about the study and, if she is still interested in participating, make an appointment for a home visit.<br>Information about the study and the informed consent form (ICF) are sent by e-mail to the potential participant. During the home visit the study will be further explained, the ICF will be signed, biochemical validations (urinary cotinine level, CO breath test and/or PEth test to validate smoking and/or alcohol use among potential participants) will be performed as indicated, and a cessation plan will be made with the researcher (LB). A quit date will be set within two weeks after the home visit, potentially difficult moments will be discussed, and a plan to avoid or deal with these moments will be devised by the participant. Using motivational interviewing, motivation and self-confidence will be increased.
Ethics oversight	The Medical Research Ethics Committee of the Erasmus MC approved the study (NL67428.078.18).

Note that full information on the approval of the study protocol must also be provided in the manuscript.

Clinical data

Policy information about clinical studies

All manuscripts should comply with the ICMJE guidelines for publication of clinical research and a completed CONSORT checklist must be included with all submissions.

Clinical trial registration	Trial NL7493
Study protocol	This paper is a protocol paper.
Data collection	Participants receive questionnaires through e-mail and fill them out online. They receive a voucher worth 15 euros if they filled out the questionnaire. Participants receive a questionnaire at inclusion and one week before each group session. Women who are pregnant during the study period also receive a questionnaire at 34 to 38 weeks gestation and a final questionnaire one week postpartum. Women who do not become pregnant during the study period receive their final questionnaire after the sixth group session. In addition, a log on patient recruitment, collaboration with local entrepreneurs, and contact with healthcare providers and participants will be kept by the research team to explore barriers and facilitators for implementation of the intervention. In the log quantitative data will be noted, such as the number of potential participants referred and included and the amount of incentives provided (outcome measures are shown in table 3). In addition, participants will fill out questionnaires about their experiences with the intervention and study itself during their participation and at the end of their participation. These data will give insight into acceptability and feasibility of the SAFER pregnancy intervention and therefore potential barriers and facilitators for proper implementation can be identified. These potential barriers and facilitators will be further qualitatively evaluated in a focus group study at the end of the SAFER pregnancy study, with the aim to evaluate the acceptability and feasibility of the intervention, in three separate focus groups with participants, leaders of the group sessions and involved healthcare providers.<br><br>At inclusion, smoking will be confirmed with a hand-held CO monitor (Micro+ smokerlyzer) and a urinary cotinine test,and alcohol use via a PhosphatidylEthanol (PEth) test in 10 mls of blood. The cut-off value to identify active (as distinguished from passive) smoking in our study will be seven ppm, in accordance with the NICE guideline. Urinary cotinine values above 50 µg/L (corrected for creatinine) will be considered indicative of active (distinguished from passive) smoking. Whole blood PEth values above 6 µg/L will be considered indicative of alcohol use in the previous two weeks. If participants report smoking cessation during the study period, this will be validated with the CO test. At the primary endpoint, smoking cessation will be validated with the CO test and the urinary cotinine test. Cessation of alcohol will be validated with the PEth test. We started recruiting participants for our study on the 1st of March 2019.
Outcomes	The primary outcome is biochemically validated cessation of smoking or alcohol use at (Figure 1):<br>- week 34 to 38 of gestation (if pregnant at inclusion or became pregnant during participation); or<br>- the end of the project period if <34 weeks pregnant at the time; or<br>- the last validation after six group sessions in those who did not become pregnant during participation.<br><br>The secondary outcomes:<br>- Barriers and facilitators of implementation<br>- Perceived efficiency and appreciation of the web-based platform, the group sessions, and incentives<br>- Costs<br>- Pregnancy outcomes

ChIP-seq

Data deposition

- [ ] Confirm that both raw and final processed data have been deposited in a public database such as GEO.
- [ ] Confirm that you have deposited or provided access to graph files (e.g. BED files) for the called peaks.

Data access links<br>*May remain private before publication.*	*For "Initial submission" or "Revised version" documents, provide reviewer access links. For your "Final submission" document, provide a link to the deposited data.*
Files in database submission	*Provide a list of all files available in the database submission.*
Genome browser session<br>(e.g. UCSC)	*Provide a link to an anonymized genome browser session for "Initial submission" and "Revised version" documents only, to enable peer review. Write "no longer applicable" for "Final submission" documents.*

Methodology

Replicates	*Describe the experimental replicates, specifying number, type and replicate agreement.*
Sequencing depth	*Describe the sequencing depth for each experiment, providing the total number of reads, uniquely mapped reads, length of reads and whether they were paired- or single-end.*
Antibodies	*Describe the antibodies used for the ChIP-seq experiments; as applicable, provide supplier name, catalog number, clone name, and lot number.*
Peak calling parameters	*Specify the command line program and parameters used for read mapping and peak calling, including the ChIP, control and index files used.*
Data quality	*Describe the methods used to ensure data quality in full detail, including how many peaks are at FDR 5% and above 5-fold enrichment.*
Software	*Describe the software used to collect and analyze the ChIP-seq data. For custom code that has been deposited into a community repository, provide accession details.*

Flow Cytometry

Plots

Confirm that:

- [ ] The axis labels state the marker and fluorochrome used (e.g. CD4-FITC).
- [ ] The axis scales are clearly visible. Include numbers along axes only for bottom left plot of group (a 'group' is an analysis of identical markers).
- [ ] All plots are contour plots with outliers or pseudocolor plots.
- [ ] A numerical value for number of cells or percentage (with statistics) is provided.

Methodology

Sample preparation	*Describe the sample preparation, detailing the biological source of the cells and any tissue processing steps used.*
Instrument	*Identify the instrument used for data collection, specifying make and model number.*
Software	*Describe the software used to collect and analyze the flow cytometry data. For custom code that has been deposited into a community repository, provide accession details.*
Cell population abundance	*Describe the abundance of the relevant cell populations within post-sort fractions, providing details on the purity of the samples and how it was determined.*
Gating strategy	*Describe the gating strategy used for all relevant experiments, specifying the preliminary FSC/SSC gates of the starting cell population, indicating where boundaries between "positive" and "negative" staining cell populations are defined.*

- [ ] Tick this box to confirm that a figure exemplifying the gating strategy is provided in the Supplementary Information.

Magnetic resonance imaging

Experimental design

Design type	*Indicate task or resting state; event-related or block design.*

Design specifications	*Specify the number of blocks, trials or experimental units per session and/or subject, and specify the length of each trial or block (if trials are blocked) and interval between trials.*
Behavioral performance measures	*State number and/or type of variables recorded (e.g. correct button press, response time) and what statistics were used to establish that the subjects were performing the task as expected (e.g. mean, range, and/or standard deviation across subjects).*

Acquisition

Imaging type(s)	*Specify: functional, structural, diffusion, perfusion.*
Field strength	*Specify in Tesla*
Sequence & imaging parameters	*Specify the pulse sequence type (gradient echo, spin echo, etc.), imaging type (EPI, spiral, etc.), field of view, matrix size, slice thickness, orientation and TE/TR/flip angle.*
Area of acquisition	*State whether a whole brain scan was used OR define the area of acquisition, describing how the region was determined.*

Diffusion MRI ☐ Used ☐ Not used

Preprocessing

Preprocessing software	*Provide detail on software version and revision number and on specific parameters (model/functions, brain extraction, segmentation, smoothing kernel size, etc.).*
Normalization	*If data were normalized/standardized, describe the approach(es): specify linear or non-linear and define image types used for transformation OR indicate that data were not normalized and explain rationale for lack of normalization.*
Normalization template	*Describe the template used for normalization/transformation, specifying subject space or group standardized space (e.g. original Talairach, MNI305, ICBM152) OR indicate that the data were not normalized.*
Noise and artifact removal	*Describe your procedure(s) for artifact and structured noise removal, specifying motion parameters, tissue signals and physiological signals (heart rate, respiration).*
Volume censoring	*Define your software and/or method and criteria for volume censoring, and state the extent of such censoring.*

Statistical modeling & inference

Model type and settings	*Specify type (mass univariate, multivariate, RSA, predictive, etc.) and describe essential details of the model at the first and second levels (e.g. fixed, random or mixed effects; drift or auto-correlation).*
Effect(s) tested	*Define precise effect in terms of the task or stimulus conditions instead of psychological concepts and indicate whether ANOVA or factorial designs were used.*

Specify type of analysis: ☐ Whole brain ☐ ROI-based ☐ Both

Statistic type for inference<br>(See Eklund et al. 2016)	*Specify voxel-wise or cluster-wise and report all relevant parameters for cluster-wise methods.*
Correction	*Describe the type of correction and how it is obtained for multiple comparisons (e.g. FWE, FDR, permutation or Monte Carlo).*

Models & analysis

n/a	Involved in the study
☐	☐ Functional and/or effective connectivity
☐	☐ Graph analysis
☐	☐ Multivariate modeling or predictive analysis

Functional and/or effective connectivity	*Report the measures of dependence used and the model details (e.g. Pearson correlation, partial correlation, mutual information).*
Graph analysis	*Report the dependent variable and connectivity measure, specifying weighted graph or binarized graph, subject- or group-level, and the global and/or node summaries used (e.g. clustering coefficient, efficiency, etc.).*
Multivariate modeling and predictive analysis	*Specify independent variables, features extraction and dimension reduction, model, training and evaluation metrics.*
